# P70 - Testing adherence to asthma clinical guidelines in Spain

**DOI:** 10.1186/2045-7022-4-S1-P125

**Published:** 2014-02-28

**Authors:** Leticia Gonzalez-Martin, Fernando Centeno-Malfaz, Roberto Velasco-Zuñiga, Juan Enrique Trujillo Wurttele, Jose Luis Fernández Arribas, Sara Puente-Montes, Maria Nathalie Campo-Fernández, Sara Martin-Armentia, Rebeca Mozun-Rico, Elena Perez-Gutiérrez

**Affiliations:** 1 Rio Hortega Hospital, Valladolid, Spain

## Objectives

To grade clinical practice variation within pediatricians of the north region of Spain society (SCCALP), and the adherence to current evidenced-based clinical guidelines.

To test significant differences depending on the grade of experience and work environment.

## Materials and methods

An anonymous survey was emailed to the pediatrician members of SCCALP using the online platform provided by Google Drive. The test consisted in multiple clinical scenarios, and multiple answers were available.

## Results

A total of ninety-nine surveys were submitted. Distribution of the pediatricians who answered was as follow: 15% ER, 14% inpatient ward, 10% subspecialties, 6% neonatology, 41% general pediatrics, and 13% were residents.

On a clinical scenario of an asthma exacerbation, 71,8% pediatricians correctly identified the severity of the exacerbation as moderate using the pulmonary score, and 86,2% would start an oral glucocorticosteroids treatment plus inhaled rapid-acting b2 agonists at adequate doses. Moreover, up to 48,9% would start a controller treatment. However, only 89,2% of them would use an adequate inhaled glucocorticosteroids dose and for an adequate period of time.

There were no significant differences in the population surveyed answers, although their experience and working environment were not homogeneous.

## Conclusions

Severity scores are not widespread in our region, although this fact does not affect the assessment and correct treatment of exacerbations.

There is a positive trend towards starting a controller treatment from the ER consultation.

The adherence to the current asthma evidence-based clinical guidelines is insufficient.

It is important to perform periodical revisions of the adherence to the guidelines in order to detect our flaws and to improve our clinical performance.

